# The role of vascular factors in Alzheimer's disease: from cerebrovascular pathological mechanisms to blood biomarkers

**DOI:** 10.3389/fnagi.2026.1997082

**Published:** 2026-10-28

**Authors:** Degang Luo, Xiangwei Lv, Huaiqian Mu, Xuele Tao, Hang Chen, Zhikang Cui, Peixian Gao

**Affiliations:** 1 Department of Vascular Surgery, Shandong Provincial Hospital affiliated to Shandong First Medical University, Jinan, Shandong, China; 2 Department of Oncology, Shandong Provincial Hospital affiliated to Shandong First Medical University, Jinan, Shandong, China; 3 Department of Breast and Thyroid Surgery, Shandong Provincial Hospital affiliated to Shandong First Medical University, Jinan, Shandong, China; 4 Department of Clinical Laboratory, Shandong Provincial Hospital affiliated to Shandong First Medical University, Jinan, Shandong, China

**Keywords:** Alzheimer's disease, blood biomarkers, blood-brain barrier, carotid stenosis, cerebral small vessel diseases, cerebrovascular trauma, glial fibrillary acidic protein, neurovascular coupling

## Abstract

Alzheimer's disease (AD) is the most common dementia, and post-mortem and imaging studies consistently demonstrate that vascular pathology coexists with plaques and tangles in most AD brains. Vascular risk factors—hypertension, diabetes, dyslipidaemia, and atrial fibrillation—damage the neurovascular unit, disrupt the blood-brain barrier, and induce chronic hypoperfusion, thereby promoting β-amyloid (Aβ) deposition, tau hyperphosphorylation, and neuroinflammation. Cerebral small vessel disease, amyloid angiopathy, and carotid stenosis are overrepresented in AD and accelerate cognitive decline. Ultrasensitive blood assays now enable plasma Aβ42/40, phosphorylated tau isoforms, glial fibrillary acidic protein, and neurofilament light chain to approach the diagnostic accuracy of cerebrospinal fluid and PET, while endothelial injury markers such as vascular endothelial-cadherin and soluble adhesion molecules are emerging. Against this background, this review contends that AD diagnostics must move beyond a single-pathology framework toward an integrated model that combines neurodegenerative and vascular injury biomarkers. Reliance on Aβ and tau alone cannot capture clinical heterogeneity nor guide vascular-targeted interventions. A multidimensional blood panel reflecting pericyte loss, endothelial dysfunction, and blood-brain barrier compromise may offer improved stratification and ultimately help identify whether a biomarker-defined subgroup could benefit from vascular interventions—a hypothesis that remains to be tested prospectively. This review traverses the entire cascade from risk factors through cerebrovascular mechanisms to surgical considerations and blood biomarkers, aiming to provide a clinically actionable reference for multidisciplinary dementia care.

## 1 Introduction

Alzheimer's disease is the leading cause of dementia in the elderly. Its global burden continues to rise as populations age. The classic amyloid cascade and tau hypotheses explain the formation of extracellular Aβ plaques and intracellular neurofibrillary tangles. However, these models cannot account for AD's highly heterogeneous clinical presentations. They also fail to address its deep entanglement with cerebrovascular pathology. Two decades ago, de la Torre asked whether AD is a neurodegenerative or a vascular disorder. That question still lacks a simple answer (de la Torre, 2004). Iadecola's account of the pathobiology of vascular dementia likewise stressed the broad overlap and synergy between vascular and neurodegenerative mechanisms (Iadecola, 2013). Since then, accumulating epidemiological, neuropathological, and neuroimaging evidence has largely settled on one judgment: multiple aspects of vascular aging, including arteriosclerosis, endothelial dysfunction, blood–brain barrier (BBB) leakage, and impaired cerebral blood flow (CBF) regulation, can independently or synergistically drive AD pathology forward (Cortes-Canteli and Iadecola, 2020). AD and vascular dementia (VD) overlap heavily at the levels of pathology, clinical features, and risk factors, and how to differentiate and jointly assess them has become an unavoidable issue in modern dementia care (Hurla et al., 2025). A 2025 scientific statement from the American Heart Association (AHA) put it plainly: the population-attributable contribution of vascular factors to cognitive impairment and dementia, including AD, should not be underestimated, and the prevalence and incidence of vascular cognitive impairment and dementia caused by vascular lesions are rising among older Americans (Smith et al., 2025).

The relationship between cerebrovascular and AD pathology is not a simple one-plus-one (He et al., 2020). Vascular changes in the brain can participate directly in the initiation and amplification of Aβ and tau pathology by lowering amyloid clearance efficiency and aggravating energy metabolism disorders and neuroinflammation (Iadecola and Gottesman, 2018); the mechanistic network through which vascular risk factors become linked to AD molecular pathology, via BBB disruption and metabolic syndrome, has likewise been systematically mapped (He et al., 2020). For laboratory diagnostics, these mechanisms imply a shift in diagnostic direction (Jack et al., 2018, 2024). AD can no longer be diagnosed solely by CSF or imaging. The NIA-AA 2018 research framework introduced the AT(N) classification—amyloid (A), tau (T), and neurodegeneration (N)—as a biological definition of AD, and the 2024 revised criteria refined this system by folding neurodegeneration into the tau category and incorporating additional biomarker axes for inflammation/immune mechanisms (I), α-synucleinopathy (S), and vascular brain injury (V). The inclusion of vascular brain injury as a formal biomarker category in the 2024 framework represents a significant conceptual advance, acknowledging that cerebrovascular pathology is not merely a comorbid condition but a biologically meaningful dimension of the AD continuum. Building on this foundation, our review argues that blood-based markers of vascular injury merit systematic integration into the diagnostic algorithm as a complement to core amyloid and tau markers. Blood biomarkers reflect two broad categories of processes: neurodegeneration and vascular injury. Together, they may piece together a more complete picture. This supports early diagnosis and risk stratification. This review follows that thread. It begins with vascular risk factors. It then addresses cerebrovascular pathological mechanisms. It also discusses carotid stenosis and its surgical interventions. Finally, it focuses on advances in laboratory diagnostics centered on blood biomarkers.

Based on the evidence summarised below, this review advances a central proposition: the diagnostic workup for AD can no longer treat vascular pathology as a secondary comorbidity. Instead, a combined assessment of amyloidosis, tauopathy, and neurovascular unit integrity—quantifiable through emerging blood biomarkers—should become the diagnostic standard. Critically, this integrated strategy may also inform future research into whether vascular interventions can modify cognitive trajectories in biomarker-defined subgroups—though the largest randomised evidence to date (CREST-2/CREST-H) has not demonstrated a cognitive benefit from carotid revascularisation. Whether biomarker-based stratification can identify patients whose cognitive impairment is predominantly haemodynamic rather than neurodegenerative remains an open research question. Accordingly, the following sections trace the pathway from conventional vascular risk factors through cerebrovascular pathogenic cascades to the latest blood biomarker developments, with the overarching goal of outlining a biologically grounded framework for early identification, stratification, and targeted management.

## 2 Epidemiological associations between vascular risk factors and AD

The concept of vascular cognitive impairment (VCI) spans the entire spectrum from vascular risk factors to overt dementia. The definition given in Nature Reviews Disease Primers is unambiguous. VCI is defined as cognitive impairment caused or aggravated by cerebrovascular disease. Management of vascular risk factors is the most important modifiable target for preventing VCI and dementia (van der Flier et al., 2018). Figure 1 summarizes the multi-step cascade by which systemic vascular risk factors drive AD pathogenesis, from hypoperfusion and BBB breakdown through neuroinflammation and impaired Aβ clearance to plaque and tangle formation and, ultimately, cognitive decline (Chun et al., 2023). Importantly, hypertension, BPV, and AF are robustly associated with all-cause dementia and vascular cognitive impairment; their association with confirmed AD neuropathologic change is less consistent. Midlife hypertension and BPV have been linked to AD pathology in some cohorts, but many studies use clinical dementia diagnosis without biomarker or autopsy confirmation. We therefore distinguish dementia risk from AD-specific pathology throughout this section. In a cohort of 205 individuals classified by the AT(N) framework, Chun et al. found that among amyloid-positive (A+) participants, those with concomitant vascular burden (V+) had a significantly lower frequency of AD (A + T+) than those without vascular burden (31.8% vs 64.4%, p = 0.004), yet showed more severe cognitive trajectories. These findings suggest that vascular burden contributes to cognitive impairment independently of, and in addition to, amyloid and tau pathology.

**Figure 1:**
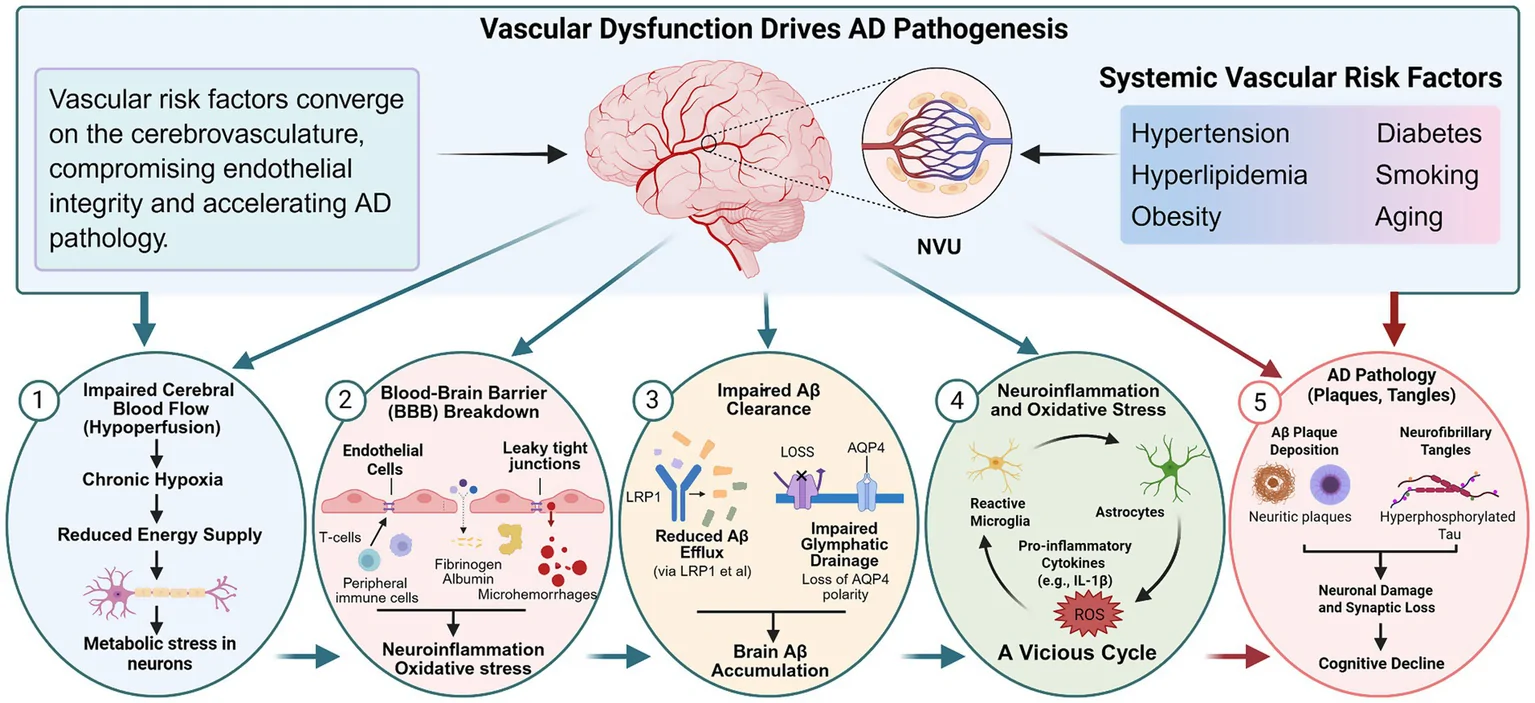
A schematic illustration of how systemic vascular risk factors drive Alzheimer’s disease pathogenesis via a multi-step cascade. Vascular risk factors lead to hypoperfusion and BBB breakdown, initiating a cascade of chronic hypoxia, metabolic stress, and neuroinflammation. These early events impair Aβ clearance mechanisms, resulting in brain Aβ accumulation. The subsequent neuroinflammatory vicious cycle further exacerbates oxidative stress, ultimately promoting the formation of plaques and tangles, which culminates in neuronal damage, synaptic loss, and cognitive decline (Created with BioRender.com). AD, Alzheimer's disease; BBB, blood-brain barrier; Aβ, amyloid-beta; LRP1, lipoprotein receptor-related protein 1; AQP4, aquaporin-4; ROS, reactive oxygen species; IL-1β, interleukin-1 beta; NVU, neurovascular unit.

### 2.1 Hypertension

A systematic review and meta-analysis of 209 prospective studies showed that hypertension was significantly associated with cognitive impairment and all-cause dementia, including vascular cognitive impairment. Midlife hypertension was especially dangerous (Ou et al., 2020). Hypertension injures the brain through multiple pathways. With blood pressure persistently elevated, vascular remodeling, arteriosclerosis, and endothelial dysfunction follow in succession; CBF autoregulation deteriorates, and BBB permeability rises (Pacholko et al., 2024); the condition can also directly impair neurovascular coupling through neuroinflammation, oxidative stress, and dysfunction of the perivascular space (PVS), dragging synapses and neurons down with it (Santisteban et al., 2023). Recent studies have added another angle. Blood pressure variability (BPV), rather than mean blood pressure alone, may be a more sensitive predictor of cognitive impairment; the amplitude of visit-to-visit blood pressure fluctuations is positively correlated with the progression of cerebral infarction, AD pathology, and cognitive decline (Ma et al., 2021).

### 2.2 Diabetes and insulin resistance

The concept of brain insulin resistance holds that cerebral insulin signaling can go awry. This leads to reduced Aβ clearance, tau hyperphosphorylation, diminished synaptic plasticity, and neuroinflammation. These events bridge diabetes and AD (Arnold et al., 2018). On one hand, diabetes injures vessels directly through microvascular lesions, including basement membrane thickening and endothelial dysfunction; on the other, it adds fuel through metabolic disturbances such as advanced glycation end products and oxidative stress. The risk of dementia in diabetic patients is consequently significantly higher than in the general population.

### 2.3 Atrial fibrillation and other risk factors

The link between atrial fibrillation (AF) and the risk of all-cause dementia and vascular cognitive impairment rests on three pathways: hypoperfusion, microembolism, and inflammation. Cardioembolism causes subclinical infarction. Fluctuations in cardiac output produce cerebral hypoperfusion. Systemic inflammation damages the BBB (Varrias et al., 2024). In addition, traditional cardiovascular risk factors such as dyslipidemia, smoking, obesity, and physical inactivity have all been confirmed to participate in AD pathogenesis (van der Flier et al., 2018). Controlling these factors is central to a brain-heart co-management preventive strategy, whose public health significance for reducing the all-cause dementia and vascular cognitive impairment burden is hard to overstate. Table 1 summarizes the epidemiological evidence linking major vascular risk factors to AD/dementia.

**Table 1:** Epidemiological evidence linking major vascular risk factors to AD/dementia.

Risk factor	Main mechanisms	Representative evidence	References
Hypertension	Vascular remodeling, endothelial dysfunction, impaired cerebral blood flow autoregulation, increased BBB permeability	Meta-analysis of 209 prospective studies: significantly associated with cognitive impairment and dementia risk, with a stronger midlife effect	Ou et al. (2020); Santisteban et al. (2023); Pacholko et al. (2024)
Diabetes/insulin resistance	Brain insulin resistance, reduced β-amyloid clearance, tau hyperphosphorylation, decreased synaptic plasticity	Impaired cerebral insulin signaling is the bridge linking diabetes and AD	Arnold et al. (2018)
Atrial fibrillation	Cardioembolism, cerebral hypoperfusion, systemic inflammation damaging the BBB	Associated with increased dementia and AD risk	Varrias et al. (2024)
Blood pressure variability	Subclinical infarction, progression of neuropathology	Visit-to-visit blood pressure variability associated with cognitive decline and AD pathology progression	Ma et al. (2021)

AD, Alzheimer's disease; BBB, blood-brain barrier.

## 3 Cerebrovascular pathological mechanisms: neurovascular unit, blood-brain barrier, and chronic cerebral hypoperfusion

The neurovascular unit (NVU) is a dynamic functional complex in the brain. It comprises neurons, astrocytes, pericytes, endothelial cells, and the basement membrane. The NVU regulates CBF, BBB barrier function, and neurovascular coupling (Zlokovic, 2011). Reduced CBF and neurovascular uncoupling are already visible in the early stages of AD, preceding overt neurodegeneration (Kisler et al., 2017). Among NVU members, pericytes play a particularly critical role. Pericytes cover cerebral capillaries more extensively than any other cell type in the human body, and they regulate angiogenesis, BBB formation, and CBF homeostasis; pericyte degeneration in AD brains is strikingly linked to Aβ deposition (Sweeney et al., 2016).

BBB disruption is consistently associated with AD pathology, and BBB breakdown has been reported in AD and other neurodegenerative disorders. A leaking BBB lets blood components such as fibrinogen and coagulation factors enter the brain parenchyma. This activates neuroinflammation and impairs neuronal function (Sweeney et al., 2018). Structurally, the BBB changes in AD include loss of tight junction proteins, basement membrane thickening, and pericyte loss. Leukocytes can also cross the BBB to infiltrate the brain (Zenaro et al., 2017). Multiple drivers of BBB dysfunction include chronic inflammation, oxidative stress, and impaired pericyte-endothelial interaction in aging and neurodegeneration, making the BBB a therapeutic target (Andjelkovic et al., 2023). Figure 2 illustrates structural and functional alterations in the blood-brain barrier in AD, contrasting intact tight junctions and selective nutrient transport under healthy conditions with the physical breakdown, impaired Aβ clearance, and increased toxin entry that characterize the AD brain.

**Figure 2:**
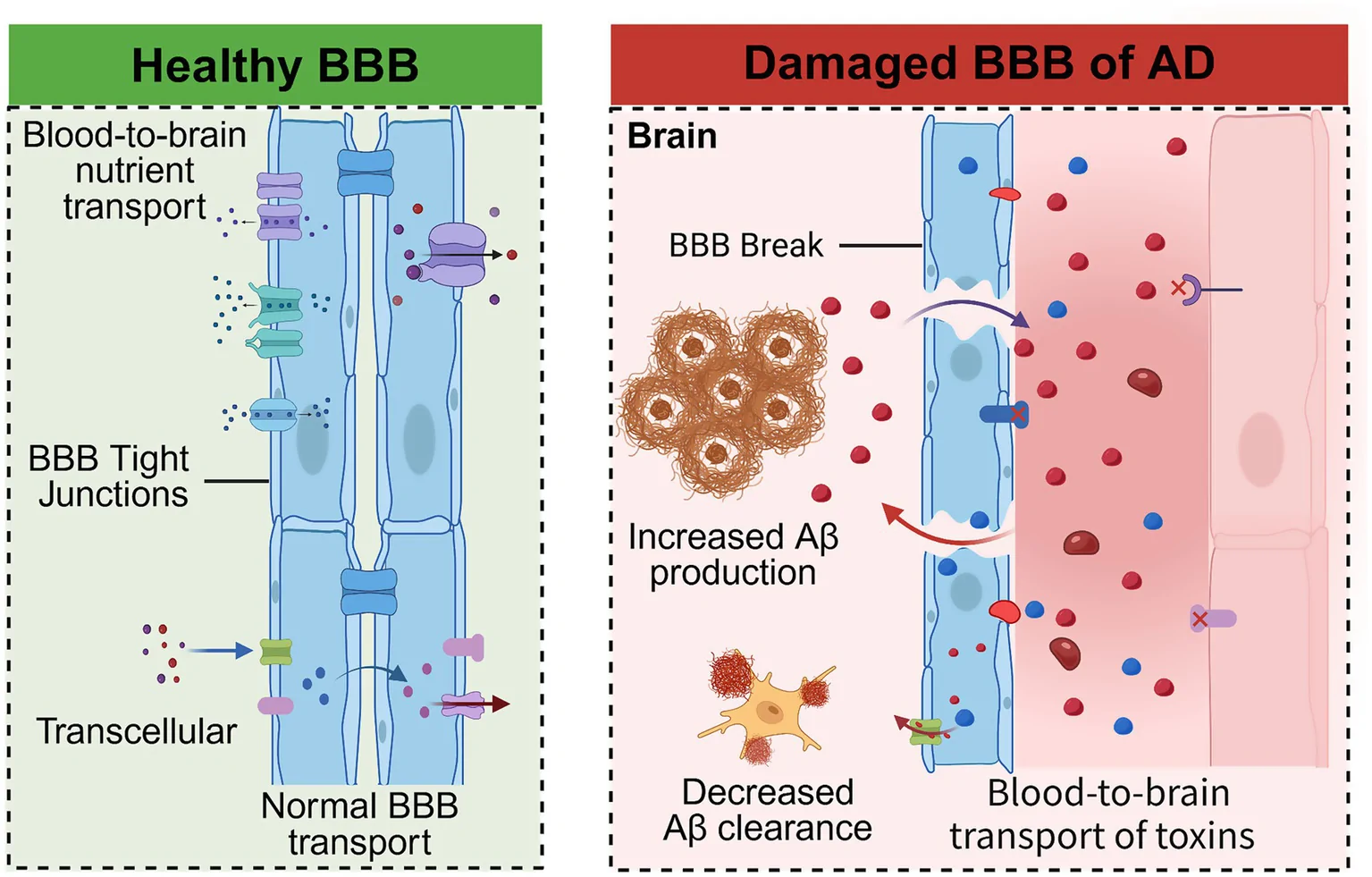
Structural and functional alterations of the blood-brain barrier in Alzheimer's disease. Under healthy conditions, the blood-brain barrier maintains cerebral homeostasis through intact tight junctions and selective transcellular nutrient transport. In Alzheimer's disease, the blood-brain barrier sustains significant damage, leading to a physical break and the disruption of normal transport pathways. This pathological progression facilitates blood-to-brain transport of toxins, increases amyloid-beta production, and decreases amyloid-beta clearance, ultimately exacerbating Alzheimer's disease pathology (Created with BioRender.com). AD, Alzheimer's disease; Aβ, amyloid-beta; BBB, blood-brain barrier.

Dynamic contrast-enhanced MRI (DCE-MRI) studies show that BBB permeability in the hippocampus and whole brain is significantly increased in AD patients. This increase is relative to cognitively normal individuals. It also correlates with vascular risk factors and AD pathology burden (Lin et al., 2021; Choi et al., 2022); in middle-aged and older adults, BBB permeability biomarkers are associated with vascular lesions and AD pathological indicators, indicating that BBB disruption can precede dementia symptoms (Edwards et al., 2025). Single-nucleus transcriptomic studies have clarified at the molecular level the transcriptional changes of vascular cells, including endothelial cells, pericytes, and smooth muscle cells, in AD brains (Sun et al., 2023), and the functional differences of each NVU component under physiological and AD conditions, together with the corresponding imaging and genetic tools, have also been systematically reviewed (Li et al., 2023). The APOE gene is the key node connecting lipid metabolism, vascular function, and AD pathology. Different APOE isoforms differentially affect BBB integrity, Aβ transport across the BBB, and pericyte survival. ε4 carriers show markedly greater BBB damage(Chen et al., 2024); APOE ε4 is not only the strongest genetic risk factor for AD but also a modifier of vascular injury (Jackson et al., 2024). Taken together, these lines of evidence point to one conclusion: NVU and BBB dysfunction have been proposed to occupy an upstream position in AD pathogenesis, and the cellular and molecular foundation of the vascular hypothesis has been substantially strengthened, although causal direction remains to be established.

Intimately linked to NVU and BBB homeostasis is the glymphatic-perivascular clearance system, which drives convective exchange between CSF and interstitial solutes. This clearance apparatus depends critically on the vasomotor pulsatility of cerebral arterioles and on aquaporin-4 polarization on astrocytic endfeet (Iliff et al., 2012; Kress et al., 2014; Chen et al., 2026). Vascular risk factors, particularly hypertension and arterial stiffening, attenuate these pulsatile driving forces, thereby prolonging solute retention within the parenchyma and reducing the washout of amyloid-β peptides (Iadecola et al., 2016). Conversely, chronic cerebral hypoperfusion further impairs glymphatic influx by diminishing cardiac-to-cerebral pulse transmission. Enlarged perivascular spaces, detectable on MRI, serve as a structural correlate of compromised clearance and have been associated with both white matter injury and cortical amyloid burden (Voumvourakis et al., 2026). Thus, the vascular-glymphatic axis constitutes an upstream rheological determinant of cerebral proteostasis, and its dysfunction may be detectable through combined imaging and fluid biomarkers before overt parenchymal deposition occurs.

Chronic cerebral hypoperfusion (CCH) is a major hemodynamic mechanism that has been implicated in vascular factor–related cognitive impairment and has been proposed as an upstream driver of NVU and BBB dysfunction. CCH denotes a long-term, sustained reduction in cerebral blood flow, a state that is often clinically silent but leaves metabolic and structural traces. Rodent models of bilateral common carotid artery stenosis have been systematically compared with human VCI/AD pathology, and CCH is considered a key link connecting vascular pathology and cognitive impairment (Duncombe et al., 2017). When cerebral blood flow falls, glucose and oxygen supply become insufficient; mitochondrial dysfunction and energy metabolism disorders ensue, which may accelerate Aβ accumulation and tau pathology in experimental models. This is the core of the energy deficit hypothesis (Love and Miners, 2016). The mechanisms by which CCH damages the BBB have been characterized mainly in animal models. Oxidative stress, inflammation, and matrix metalloproteinase activation progressively disrupt tight junctions and injure pericytes, increasing BBB permeability (Rajeev et al., 2022); CCH has been proposed to drive the etiological process of VCI through multiple vascular-metabolic-immune pathways, and carotid artery stenosis is one of the most important clinical causes of CCH (Rajeev et al., 2023). On imaging, CBF reduction measured by arterial spin labeling (ASL) MRI can serve as a marker of the preclinical stage of AD, and hypoperfusion often precedes structural atrophy (Wierenga et al., 2014); in older APOE ε4 carriers, cerebral hypoperfusion is significantly associated with impaired gray matter structural integrity and cognitive function, and genetically susceptible populations may be especially vulnerable to hypoperfusion injury (Pappas I. et al., 2025). More recently, a vascular-metabolic-immune-glymphatic network hypothesis has been proposed that integrates CCH, metabolic disorders, immune responses, and glymphatic clearance dysfunction, describing AD as the result of multi-system interactive imbalance. However, much of the evidence for CCH and glymphatic dysfunction comes from rodent models; human evidence is largely indirect, based on MRI markers such as enlarged perivascular spaces, ASL-measured CBF, and DCE-MRI. Therapeutic implications should therefore be regarded as hypothesis-generating rather than established, and the network hypothesis suggests a potential avenue for future research rather than a validated therapeutic target (Yao et al., 2026).

The strength of causal evidence in humans varies across pathways: it is strongest for midlife hypertension and later dementia, intermediate for BBB permeability and cSVD burden, and weakest for glymphatic dysfunction and endothelial injury markers. Throughout this review, we therefore distinguish associative findings from established causal evidence.

## 4 Cerebral small vessel disease and cerebral amyloid angiopathy

Cerebral small vessel disease (cSVD) is a heterogeneous group of disorders. It involves cerebral small arteries, arterioles, capillaries, and venules. Its imaging features include white matter hyperintensities (WMH), lacunar infarction, microbleeds, and enlarged perivascular spaces. On the pathophysiology of cSVD in VD and AD, a broad consensus has emerged: vascular smooth muscle cell loss, vascular fibrosis, and BBB disruption are the pathological bases shared by cSVD and AD (Inoue et al., 2023). At the risk-factor level, hypertension is the most important modifiable factor for cSVD, with a clear dose-response relationship between cSVD, hypertension, and dementia risk (Hainsworth et al., 2024).

The relationship between cSVD and AD is both close and complex. The two conditions are highly co-prevalent in the elderly. Through bidirectional vascular injury-amyloid pathology interactions, they jointly push cognitive decline deeper (Kim et al., 2020). Varying degrees of cSVD pathology are found in both sporadic and familial AD, indicating that vascular pathology runs through the entire course of AD (Kalaria and Sepulveda-Falla, 2021). A systematic review of cSVD and AD risk yields a more specific picture: the presence of cSVD significantly increases the risk of AD, particularly in individuals with coexisting vascular risk factors (Liu et al., 2018). The two conditions often appear in the same patient without either fully explaining the clinical picture, which is why the term mixed pathology has gained ground in both clinical practice and autopsy studies.

Cerebral amyloid angiopathy (CAA) is a pathological state in which Aβ is deposited in the walls of cerebral small vessels. This condition shares amyloid pathology with AD. Its pathogenesis includes impaired Aβ clearance from vessel walls and injury to vascular smooth muscle cells. Its imaging features include lobar microbleeds, cortical superficial siderosis, and white matter damage. Therapeutic advances have also been systematically reviewed (Cozza et al., 2023). In participants along the AD continuum, amyloid pathology, cSVD, and glymphatic dysfunction synergistically influence cognitive performance, and the vascular plus amyloid dual-pathology model explains clinical reality better than a single-pathology model (Hong et al., 2024). The contribution of vascular factors to AD pathology progression, with cSVD as a major component, means that vascular burden must be assessed simultaneously in the clinical diagnosis of AD, rather than treated as an optional item (Eisenmenger et al., 2023). Recent studies have found spatial associations between cSVD and tau proteinopathy and TDP-43 proteinopathy in the medial temporal lobe; small vessel lesions may promote proteinopathy accumulation by impairing protein clearance (Perosa et al., 2025). In short, cSVD is not a mere comorbidity of AD; it is a participant and modifier of the pathological process.

The in vivo characterisation of cSVD burden relies heavily on structural MRI, with white matter hyperintensities, lacunar infarcts, cerebral microbleeds, and enlarged perivascular spaces representing the core imaging phenotype (Wardlaw et al., 2013; Prins and Scheltens, 2015). Although none of these radiological findings are specific to AD, their topographic distribution and dynamic progression provide essential quantitative information about the vascular component coexisting with neurodegeneration (Graff-Radford et al., 2017; Debette et al., 2019). Lobar microbleeds favor a diagnosis of cerebral amyloid angiopathy, whereas deep or mixed microbleeds point predominantly toward hypertensive arteriopathy (Jung et al., 2020). Serial MRI captures the temporal evolution of ischaemic tissue injury, and the rate of white matter hyperintensity progression independently predicts incident dementia (de Havenon et al., 2022). These imaging metrics should be interpreted not as stand-alone diagnostic tests but as confirmatory anchors for blood-based vascular biomarker signals; for example, elevated plasma glial fibrillary acidic protein (GFAP) levels coupled with progressive white matter hyperintensities strongly argue for a vascular-predominant disease driver (Sanchez et al., 2025). We therefore contend that routine MRI-based vascular burden scoring, alongside amyloid and tau PET or fluid assays, should be incorporated into the standardised diagnostic algorithm for patients presenting with cognitive complaints.

## 5 Carotid artery stenosis and cognitive impairment: from pathological mechanisms to revascularization

Carotid artery stenosis is the predominant form of extracranial large-artery atherosclerosis. It constitutes one of the most clinically significant vascular lesions in vascular surgery. As early as the 2000s, impairments across multiple cognitive domains, including attention, executive function, and memory, were already found in patients with carotid stenosis, with hypoperfusion as the main suspect (Sztriha et al., 2009). The relationship between atherosclerotic carotid stenosis and cognitive function depends on the combined effects of stenosis severity, plaque stability, and cerebral hemodynamic changes (Wang et al., 2016). For a vascular surgeon, the clinical question is rarely whether the stenosis itself matters, but rather how much of the patient's cognitive decline it can explain and whether reopening the vessel will change the trajectory.

The relationship between asymptomatic carotid artery stenosis (ACAS) and cognitive impairment is a focus of recent debate. The systematic review commissioned by the European Society for Vascular Surgery concludes that most studies support an association between ACAS and cognitive decline, with more pronounced impairment in those with stenosis of 70% or greater. However, the level of evidence remains limited (Paraskevas et al., 2021). A meta-analysis of extracranial carotid plaque and cognitive impairment shows that plaque presence and burden are significantly associated with cognitive dysfunction (Baradaran et al., 2022). Mechanistically, carotid stenosis impairs cerebrovascular reactivity and CBF autoregulation, producing cerebral hemodynamic failure, and cognitive impairment progresses accordingly; cognitive assessment of patients with asymptomatic stenosis is therefore not optional but mandatory (Viticchi et al., 2021). The SMART-MR study also found that mild carotid stenosis is associated with progression of brain atrophy and cognitive decline, suggesting that the brain's vulnerability to carotid atherosclerosis may have no clear safe threshold (Ghaznawi et al., 2023). A 2023 review on clinical assessment strategies for cognitive impairment in ACAS patients and the indications for revascularization remained under debate (Paraskevas et al., 2023); earlier systematic reviews, while supporting the association between ACAS and cognitive decline, also called for higher-quality longitudinal studies to settle the matter (Chang et al., 2013).

A dose-response relationship deserves particular mention. Neuropsychological testing shows that the degree of stenosis itself can predict dementia (Machaczka et al., 2022). The latest large-sample observational study delivers an encouraging signal: carotid endarterectomy (CEA) in patients with asymptomatic carotid stenosis can reduce the associated risk of AD and non-AD dementia (Vitali et al., 2025). This means that surgically intervening on vascular lesions to prevent dementia is no longer merely theoretical, and it opens space for joint research among vascular surgery, neurology, and laboratory medicine.

Whether revascularization benefits cognition is one of the most debated topics in vascular surgery. Revascularization includes CEA and carotid artery stenting (CAS). A prospective randomised controlled study comparing post-procedural cognitive changes between CEA and CAS found that the patterns did differ between the two modalities. Yet, neither showed significant long-term cognitive benefit, and some patients even experienced early postoperative cognitive fluctuation (Altinbas et al., 2011). A systematic review of cognitive function after CEA in asymptomatic patients is equally cautious: current evidence is insufficient to establish whether CEA improves cognition, with high heterogeneity and methodological differences as the main confounders (Sridharan et al., 2022). Narrative reviews reach similar conclusions and call for standardized neuropsychological test protocols and longer follow-up (Piegza et al., 2021). That said, specific subgroups may genuinely benefit. CAS improves cognitive function in patients with carotid stenosis and cerebral hemodynamic impairment, and patients in hemodynamic failure are the most likely beneficiaries (Hara et al., 2020); improvement in some cognitive domains was observed at 1-year follow-up after CAS (Piegza et al., 2022). Improvement in cerebrovascular reactivity, that is, cerebrovascular regulation capacity, after CEA correlates with postoperative cognitive changes, supporting the flow restoration and cognitive improvement hypothesis. However, early postoperative microembolism and hyperperfusion may offset part of the benefit (Sándor et al., 2024). A confounding factor that previous studies generally neglected is cerebral small vessel disease burden, and future patient stratification should be based on MRI vascular burden (Arba et al., 2022). Overall, the cognitive effects of carotid intervention vary considerably among individuals, and identifying beneficiary populations, namely younger age, low cSVD burden, and presence of hemodynamic impairment, is the way forward (Succar and Zhou, 2023); a comparison of cognitive changes after CEA and CAS performed a decade apart showed no significant differences between the modalities, but early postoperative cognitive fluctuation was associated with perioperative cerebral ischemic events in both (Václavík et al., 2023). The verdict is not yet in; rigorously designed randomised controlled trials are urgently needed, and stratifying patients with imaging vascular burden plus blood biomarkers is precisely where laboratory diagnostics can engage deeply.

Synthesising the available surgical and biomarker evidence, including the CREST-2 randomised trials and the CREST-H substudy (Lazar et al., 2026; Marshall et al., 2026), a more cautious interpretation is warranted. The cognitive response to carotid revascularisation is conspicuously heterogeneous, and the largest randomised evidence to date does not support a cognitive benefit. The CREST-2 randomised trials (*n* = 2,165 across CAS and CEA arms) found no significant difference in cognitive outcomes between revascularisation plus intensive medical management and medical management alone over a mean follow-up of 2.2 years. Critically, the CREST-H substudy (*n* = 291 analysed) specifically tested whether patients with baseline cerebral hemodynamic impairment derive differential cognitive benefit; no such benefit was observed at one year, regardless of hemodynamic status. These findings suggest that restoring cerebral blood flow may not reverse cognitive impairment once chronic asymptomatic carotid stenosis is established, and that the assumption of a direct cognitive benefit from revascularisation is not validated by current randomised evidence.

This does not diminish the biological plausibility of a vascular contribution to cognitive decline. Rather, it reframes the clinical question: whether a biomarker-defined subgroup—characterised by haemodynamically significant stenosis, low core Alzheimer’s pathology, and limited cSVD burden—might selectively benefit remains an open research question. The cognitive endpoints in CREST-2 were secondary outcomes and the follow-up duration may be insufficient to detect small effects; moreover, patients were enrolled without biomarker-based stratification. Prospective validation in biomarker-stratified surgical cohorts is therefore needed before any cognitive indication for revascularisation can be considered. At present, revascularisation should continue to be indicated for stroke prevention on anatomical grounds, and its cognitive effects should be framed as a hypothesis-generating research question rather than a clinical recommendation.

## 6 Blood biomarkers: laboratory progress of core AD markers

Blood biomarkers are the brightest breakthrough in AD laboratory diagnosis in recent years (Ashton et al., 2024). The development of ultrasensitive immunoassays such as the single-molecule array (Simoa) and mass spectrometry (LC-MS/MS) has enabled stable detection of trace amounts of Aβ and tau protein isoforms in plasma, and blood testing is moving from a research tool toward clinical laboratory testing (Ashton et al., 2024; Zeng et al., 2024). For the clinical laboratory, the appeal is obvious: a venous blood draw is far less invasive than a lumbar puncture (van Gennip et al., 2024), far cheaper than an amyloid PET scan (Woo et al., 2024), and repeatable across the course of the disease (Raghavan et al., 2025). Figure 3 depicts the dynamic trajectories of blood-based biomarkers across the AD continuum, mapping each biomarker to its underlying pathological event, from early amyloid pathology and astrogliosis to axonal injury and, concurrently, vascular dysfunction within the neurovascular unit.

**Figure 3:**
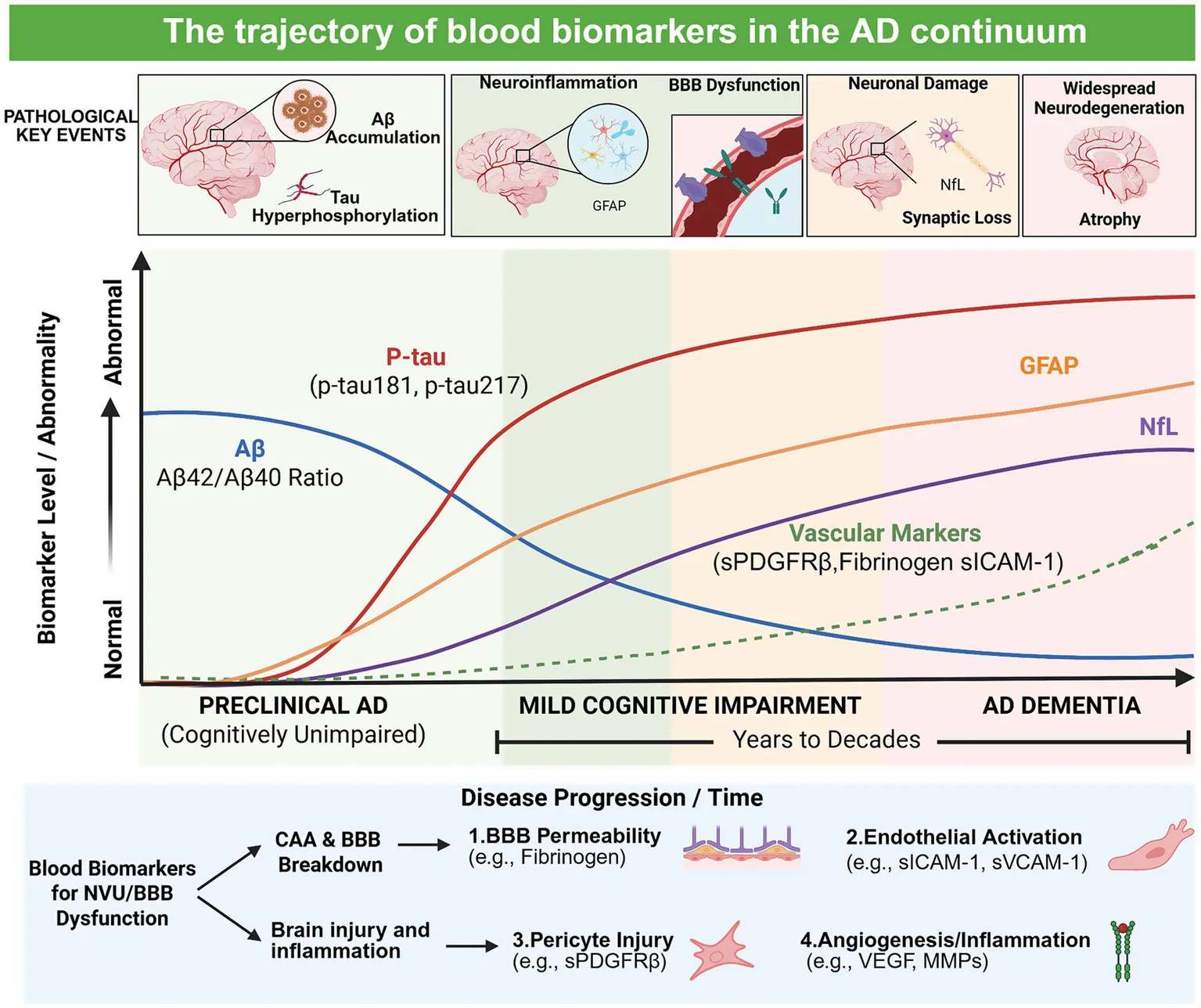
Schematic overview of blood biomarker trajectories across the Alzheimer’s disease continuum. The figure correlates key pathological events with the dynamic changes in blood-based biomarkers during disease progression. Amyloid-beta pathology begins in the preclinical stage, followed by increases in p-tau and GFAP, indicating tau pathology and astrogliosis. As the disease advances to mild cognitive impairment and dementia, NfL increases, reflecting axonal and neuronal damage. Concurrently, vascular markers emerge, tracking the progression of BBB dysfunction, pericyte injury, and endothelial activation within the neurovascular unit. The bottom panel specifically maps blood biomarkers to distinct vascular pathologies, including BBB permeability, endothelial activation, pericyte injury, and angiogenesis (Created with BioRender.com). AD, Alzheimer’s disease; Aβ, amyloid-beta; BBB, blood-brain barrier; CAA, cerebral amyloid angiopathy; GFAP, glial fibrillary acidic protein; MCI, mild cognitive impairment; NfL, neurofilament light chain; NVU, neurovascular unit; p-tau, phosphorylated tau; sICAM-1, soluble intercellular adhesion molecule-1; sPDGFRβ, soluble platelet-derived growth factor receptor-beta; sVCAM-1, soluble vascular cell adhesion molecule-1; VEGF, vascular endothelial growth factor; MMPs, matrix metalloproteinases.

### 6.1 Plasma Aβ42/40 ratio

The plasma Aβ42/40 ratio is the classic biomarker reflecting cerebral amyloid pathology. Cross-sectional and longitudinal analyses in the AIBL cohort show that Aβ42/40 declines early in amyloid pathology and is among the earliest plasma markers to change (Chatterjee et al., 2023). The ratio has a practical advantage: because it compares two closely related peptides measured in the same sample, it is less sensitive to pre-analytical variation such as hemolysis, storage time, or batch effects than an absolute concentration would be. A 2022 multicentre validation study measured plasma Aβ42/Aβ40 by immunoprecipitation mass spectrometry (IP-MS) in 465 participants from three independent AD research cohorts (United States, *n* = 182; Australia, *n* = 183; Sweden, n = 100). Plasma Aβ42/Aβ40 showed good concordance with amyloid PET status (AUC 0.84, 95% CI 0.80–0.87), and the AUC with CSF amyloid status was 0.85 (95% CI 0.78–0.91), improving to 0.93 (95% CI 0.89–0.97) when APOE ε4 carrier status was included. These findings were consistent across the three cohorts despite differences in blood collection and processing protocols, and the assay performed similarly in cognitively unimpaired and impaired individuals (Li et al., 2022). Plasma biomarkers should not yet be described as globally replacing CSF. They can serve as triage in specialist memory clinics using validated assays, such as FDA-cleared p-tau217/Aβ42 platforms or LC-MS/MS, with two-cutoff approaches and CSF/PET confirmation for indeterminate or treatment-relevant cases. In primary care, positive predictive value is lower because of lower pre-test probability, and confirmatory testing is needed. Asymptomatic population screening remains a research setting. Validation criteria include platform-specific cut-offs, pre-analytical standardization, and prospective demonstration of clinical utility.

### 6.2 Plasma phosphorylated tau

Phosphorylated tau (p-tau) is the blood biomarker with the highest AD specificity, and p-tau217 is widely recognized as the best-performing isoform. Validation in clinical and community cohorts shows that the plasma p-tau217/Aβ42 combination effectively differentiates AD from other dementias (Wang et al., 2025); multi-ethnic community studies also confirm that p-tau181 and p-tau217 have diagnostic and screening value in non-clinical populations (Brickman et al., 2021). The attraction of p-tau231 lies in its earliness: it rises earlier than p-tau181. It can serve as a marker of incipient amyloid pathology (Ashton et al., 2021), tracking amyloid pathology status together with p-tau217 in the preclinical stage (Milà-Alomà et al., 2022). The predictive data are even more compelling. Plasma p-tau217 combined with brief cognitive tests and APOE genotype can predict future onset of AD dementia, with predictive performance approaching that of CSF (Palmqvist et al., 2021); in cognitively normal individuals, p-tau217 and Aβ42/40 can predict cerebral Aβ accumulation in advance (Janelidze et al., 2024); and combinations of multiple plasma markers can predict longitudinal cognitive decline in preclinical AD (Mattsson-Carlgren et al., 2023). A landmark head-to-head comparison in the BioFINDER-2 (*n* = 1,422) and Knight ADRC (*n* = 337) cohorts demonstrated that plasma %p-tau217 was clinically equivalent to FDA-approved CSF tests in classifying Aβ PET status (AUC 0.95–0.97 for both) and generally superior for tau-PET classification (Barthélemy et al., 2024). A practical takeaway is that the intervention window is widening: blood tests can flag disease years before symptoms force a clinic visit, changing how early diagnosis is planned. The application prospects of plasma p-tau in diagnosis and therapeutic trials, for example, monitoring the efficacy of anti-amyloid drugs, have been systematically envisioned (Gonzalez-Ortiz et al., 2023), and Aβ42/40, p-tau231, and p-tau217 play distinct roles in trial screening and disease monitoring (Ashton et al., 2022). Standardization is an unavoidable hurdle. Absolute p-tau concentrations are not interchangeable across platforms. Head-to-head comparisons of ten plasma phospho-tau assays show good inter-assay correlations but substantial differences in absolute values. Simoa and Lumipulse automated platforms also differ in analytical performance. Assay-specific cut-offs, platform-specific validation, and longitudinal use of the same assay are required. Values obtained using different platforms should not be treated as directly interchangeable (Janelidze et al., 2023); evaluation of the analytical and clinical performance of eight Simoa and Lumipulse automated platforms for p-tau181 and p-tau217 provides a basis for laboratory selection (Wojdała et al., 2024); and clinical validation of the mass spectrometry-based PrecivityAD2 blood test, which combines %p-tau217 and the Aβ42/40 ratio, demonstrates high accuracy in identifying cerebral amyloid pathology (Meyer et al., 2024).

Assay standardisation has advanced considerably. As of August 2026, four AD blood tests have received FDA clearance, including Roche’s Elecsys pTau217 (cleared August 24, 2026, for adults 55 and older) and C2N Diagnostics’ PrecivityAD2 (cleared August 20, 2026, for adults as young as 40). The Elecsys pTau217 test demonstrated 88% agreement with PET results in cognitively impaired participants and 91% in unimpaired participants across a 2,148-adult study spanning four countries. PrecivityAD2 correctly ruled in 98% of PET/CSF-positive cases and confirmed 93% of negative results in 1,142 symptomatic adults. These clearances represent a milestone in translating plasma biomarkers from research tools to clinical diagnostics(Anderer, 2026; Biessels et al., 2026). What remains to be addressed is not whether standardisation is achievable, but how to harmonize cutoffs and quality-control systems across platforms and populations.

### 6.3 Glial and neurodegeneration markers

GFAP marks astrocyte activation, and its role in AD blood biomarker research has grown rapidly in recent years. Comparative studies of plasma and CSF GFAP across the AD continuum demonstrated that plasma GFAP achieved AUCs of 0.69–0.86 for discriminating AD from controls, exceeding the performance of CSF GFAP (AUC 0.59–0.76) and rising earlier in the presymptomatic stage, likely reflecting early astrocytic reactivity while being influenced by BBB permeability and genetic background (Benedet et al., 2021). S100B, a calcium-binding protein expressed by astrocytes, has also been proposed as a marker of BBB permeability and cSVD burden, though its utility is limited by its lack of CNS specificity (Wu et al., 2024; Pappas C. et al., 2025).

Neurofilament light chain (NfL) is a general marker of axonal injury; it is not AD-specific, but it is irreplaceable for progression monitoring and differential diagnosis, for example, distinguishing frontotemporal dementia, and correlates with the rate of cognitive decline (Chatterjee et al., 2023; Mattsson-Carlgren et al., 2023). In the AGES-Reykjavik study, higher plasma NfL was associated with greater WMH volume and incident dementia over longitudinal follow-up (van Gennip et al., 2024).

Community-based data confirm that combinations of blood markers can predict all-cause dementia, making blood markers feasible for primary care and community screening. Grande et al. analysed 2,148 community-dwelling participants and found that combinations of blood markers including p-tau217, GFAP, and NfL predicted incident all-cause dementia with AUCs of 71%–83%, with positive predictive values reaching 43% when p-tau217 was combined with NfL or GFAP (Grande et al., 2025).

Novel markers of microglial activation and astrocytic reactivity, such as soluble triggering receptor expressed on myeloid cells 2 (sTREM2), also correlate with preclinical AD progression, though their incremental value beyond established markers (p-tau217, GFAP, NfL) remains to be demonstrated in prospective cohorts.

### 6.4 Molecular markers of vascular cognitive impairment and cerebral small vessel disease

Beyond core AD markers, molecular markers reflecting vascular pathology have entered the field of view. Despite substantial research effort, the molecular biomarker repertoire for VCI remains far from complete. Hosoki et al. systematically reviewed candidate markers and concluded that progress in identifying clinically actionable molecular markers for VCI and cSVD has been limited, with no single marker yet reaching routine clinical use (Hosoki et al., 2023). Endothelial injury markers include soluble intercellular adhesion molecule-1 (sICAM-1), soluble vascular cell adhesion molecule-1 (sVCAM-1), and von Willebrand factor (vWF). BBB disruption markers such as matrix metalloproteinases (MMPs) and S100B; inflammatory markers such as interleukin-6 (IL-6) and C-reactive protein (CRP); and the neurodegeneration marker NfL have all been associated with vascular pathology. However, none has demonstrated sufficient specificity or reproducibility to serve as a standalone diagnostic. Combining AD markers with vascular markers nevertheless remains the most practical path forward for differentiating and stratifying mixed dementia (Hosoki et al., 2023). Blood biomarker research for cSVD faces an awkward situation: markers related to platelet activation, endothelial dysfunction, inflammation, and glymphatic dysfunction all correlate with cSVD imaging burden, yet a single highly specific marker is still missing (Wu et al., 2024).

Endothelial dysfunction has been proposed as an early contributor to AD vascular pathology, although whether it is a primary driver or a secondary consequence of amyloid and tau pathology remains debated (Tarawneh et al., 2022; Tarawneh, 2023). Preclinical evidence shows that vascular endothelial-cadherin (VE-cadherin) is elevated in blood before symptom onset (Tarawneh et al., 2022), and CSF markers of inflammation and vascular dysfunction, including YKL-40 and cytokines, are elevated in early AD (Janelidze et al., 2018). Mechanistically, BACE1 impairs angiogenesis and endothelial function by cleaving vascular endothelial substrates such as VEGFR1 (Katusic et al., 2024), and Aβ directly upregulates VCAM-1 in cerebrovascular endothelial cells, linking amyloid pathology to endothelial inflammatory activation (Salian et al., 2024). However, these markers are not AD-specific: sVCAM-1, sICAM-1, VE-cadherin, and vWF reflect systemic endothelial activation or injury and may be elevated in atherosclerosis, diabetes, hypertension, and other inflammatory conditions. They should therefore be interpreted alongside core AD markers (Aβ42/40, p-tau217) and MRI vascular burden, not as stand-alone diagnostic tests. Prospective validation is needed before they can be used to quantify vascular pathology burden in AD.

At the imaging–biomarker interface, Sanchez et al. demonstrated that elevated plasma GFAP levels coupled with progressive white matter hyperintensities on MRI were strongly associated with a vascular-predominant disease driver across neurodegenerative and cerebrovascular disease cohorts (Sanchez et al., 2025). This finding illustrates how a non-AD-specific glial marker can gain interpretive value when combined with a vascular imaging phenotype.

Recent proteomic studies have begun to address the gap in specificity. A plasma proteome-wide analysis within the Atherosclerosis Risk in Communities (ARIC) Study cohort examined 4,877 plasma proteins in 1,508 participants and identified 13 WMH-associated proteins involved in synaptic function, endothelial integrity, and angiogenesis; 11 of these were associated with future dementia risk, and two candidate proteins—oligodendrocyte myelin glycoprotein (OMG) and neuronal pentraxin receptor (NPTXR)—emerged as top biomarkers (Gomez et al., 2024). Caro et al. further identified a proteogenomic biological fingerprint of cSVD in both CSF and plasma, providing a foundation for blood-based cSVD detection (Caro et al., 2025).

A recent World Stroke Organization scientific statement has cataloged candidate biochemical markers for vascular cognitive impairment, including vascular endothelial growth factor-A, placental growth factor, IL-6, matrix metalloproteinase-9, and cathepsin-B. The statement also highlighted that vascular disease accounts for approximately one-third of the population-attributable risk of dementia, controlling for other pathologies, with the proportion rising to 50–70% when vascular disease is considered as co-pathology. These figures substantially strengthen the case for incorporating vascular markers into routine AD diagnostic panels (Biessels et al., 2026). The 2024 NIA-AA framework’s inclusion of vascular brain injury (V) as a biomarker category provides the conceptual scaffolding for this integrated approach. What remains to be established is whether blood-based vascular markers can achieve the analytical performance required to populate this category in routine clinical practice.

It is important to distinguish AD-specific markers from nonspecific markers. Plasma Aβ42/40 and p-tau isoforms are the only blood markers that reflect AD pathology with reasonable specificity. GFAP and NfL reflect astrogliosis and axonal injury but are not AD-specific. Vascular markers reflect endothelial injury, BBB disruption, or inflammation but are also not AD-specific. A multidimensional panel should therefore not be interpreted as a simple additive score. Interpretation should integrate MRI vascular burden and pre-test probability. An AD-positive/vascular-low profile suggests AD-predominant pathology; an AD-negative/vascular-high profile suggests vascular-predominant pathology; an AD-positive/vascular-high profile suggests mixed pathology; and discordant or indeterminate results require specialist evaluation. No composite algorithm has yet been prospectively validated.

In summary, the evidence reviewed above points to a clear trajectory: the laboratory diagnosis of AD is moving beyond a single amyloid marker toward a multi-pathological-dimension combined testing system that encompasses amyloid, tau, neurodegeneration, and vascular pathology. Tables 2, 3 summarize the laboratory characteristics of core AD blood biomarkers and vascular injury markers, respectively, providing a practical reference for the design of such multidimensional panels.

**Table 2:** Core AD blood biomarkers and their laboratory characteristics.

Biomarker	Reflected pathology	Major assay platforms	Analysis and outcomes	References
Plasma Aβ42/40 ratio	Cerebral amyloid pathology	LC-MS/MS, IP-MS	Declines early in amyloid pathology; AIBL cohort cross-sectional/longitudinal analysis	Li et al. (2022);Chatterjee et al. (2023)
p-tau217	Neuronal tau pathology	Simoa, LC-MS/MS	BioFINDER-2 (*n* = 1,422) and Knight ADRC (*n* = 337): AUC 0.95–0.97 for Aβ PET, clinically equivalent to FDA-approved CSF tests	Palmqvist et al. (2021);Barthélemy et al. (2024);Janelidze et al. (2024);Wang et al. (2025)
p-tau181	Neuronal tau pathology	Simoa, Lumipulse	Multi-ethnic community study: diagnostic and screening value in non-clinical populations	Brickman et al. (2021);Janelidze et al. (2023);Wojdała et al. (2024)
p-tau231	Early amyloid pathology	Simoa	Rises in preclinical stage, earlier than p-tau181; tracks amyloid status with p-tau217	Ashton et al. (2021);Milà-Alomà et al. (2022)
GFAP	Astrocyte activation	Simoa	Plasma GFAP AUC 0.69–0.86 vs CSF 0.59–0.76 across AD continuum	Benedet et al. (2021);Mattsson-Carlgren et al. (2023)

AD, Alzheimer's disease; Aβ, β-amyloid; CSF, cerebrospinal fluid; GFAP, glial fibrillary acidic protein; IP-MS, immunoprecipitation-mass spectrometry; LC-MS/MS, liquid chromatography-tandem mass spectrometry; p-tau, phosphorylated tau; Simoa, single-molecule array.

**Table 3:** Blood biomarkers of vascular injury.

Biomarker	Pathology reflected	Analysis and outcomes	References
VE-cadherin, vWF	Endothelial disruption/activation	VE-cadherin elevated in blood at preclinical AD stage	Tarawneh et al. (2022); Hosoki et al. (2023)
sVCAM-1, sICAM-1	Endothelial inflammatory activation	Associated with cSVD imaging burden; Aβ upregulates VCAM-1 in cerebrovascular endothelium	Hosoki et al. (2023); Salian et al. (2024)
MMPs, S100B	Tight junction disruption, BBB leakage	Candidate molecular markers of VCI; associations with cSVD imaging burden reported but specificity limited	Hosoki et al. (2023); Wu et al. (2024)
IL-6, YKL-40	Neuroinflammation	Elevated in CSF/blood of early AD patients; WSO statement lists IL-6 as candidate vascular marker	Janelidze et al. (2018); Mora et al. (2024)
VEGF-A, PlGF, MMP-9, CTSB	Angiogenesis, vascular remodelling, BBB	WSO 2026 scientific statement candidate markers for vascular contribution to dementia	Biessels et al. (2026)

BBB, blood-brain barrier; CTSB: cathepsin-B, IL-6, interleukin-6; MMP-9: matrix metalloproteinase-9, MMPs, matrix metalloproteinases; PlGF: placental growth factor, sICAM-1, soluble intercellular adhesion molecule-1; sVCAM-1, soluble vascular cell adhesion molecule-1; VE-cadherin, vascular endothelial-cadherin; VEGF-A: vascular endothelial growth factor-A, vWF, von Willebrand factor; YKL-40, chitinase-3-like protein 1 (also known as CHI3L1).

## 7 Conclusions and perspectives

In summary, vascular factors contribute substantially to AD initiation and progression through neurovascular unit dysfunction, BBB disruption, chronic cerebral hypoperfusion, cSVD, and carotid stenosis, interacting synergistically with amyloid and tau pathology. Blood biomarker research has reached a translational milestone, with plasma Aβ42/40, p-tau217, GFAP, and NfL approaching CSF- and PET-level diagnostic accuracy, and four tests now FDA-cleared for clinical use. Endothelial injury and cSVD-related markers capture the vascular dimension but require further validation.

However, blood-based biomarker results should be interpreted with caution. Not all circulating markers are CNS-derived: NfL and GFAP have peripheral sources, and endothelial markers such as VE-cadherin, sVCAM-1, and vWF reflect systemic vascular beds. BBB integrity, renal clearance, pre-analytical variables, assay platform differences, and demographic and clinical covariates all influence plasma concentrations independently of cerebral pathology. Moreover, only plasma Aβ42/40 and p-tau isoforms are AD-specific; GFAP, NfL, and vascular markers are not. A multidimensional panel therefore cannot be interpreted as a simple additive score; integration with clinical context, MRI vascular burden, and confirmatory testing is essential. Future studies should report assay platform, pre-analytical protocols, and covariate adjustment, and validate panels in prospective, biomarker-stratified cohorts.

Integrating neurodegenerative and vascular injury markers into a multidimensional blood testing system is the logical next step for laboratory diagnosis of AD, and imaging-based vascular burden assessment provides complementary information for patient stratification. Figure 4 outlines how such multidimensional panels may inform clinical decisions across primary care, memory clinics, and preclinical high-risk cohorts. Whether vascular interventions, including carotid revascularization, can modify cognitive trajectories in biomarker-defined subgroups remains an open research question—the largest randomised evidence to date (CREST-2/CREST-H) has not demonstrated a cognitive benefit. Prospective validation in biomarker-stratified surgical cohorts is needed before considering any cognitive indication.

**Figure 4:**
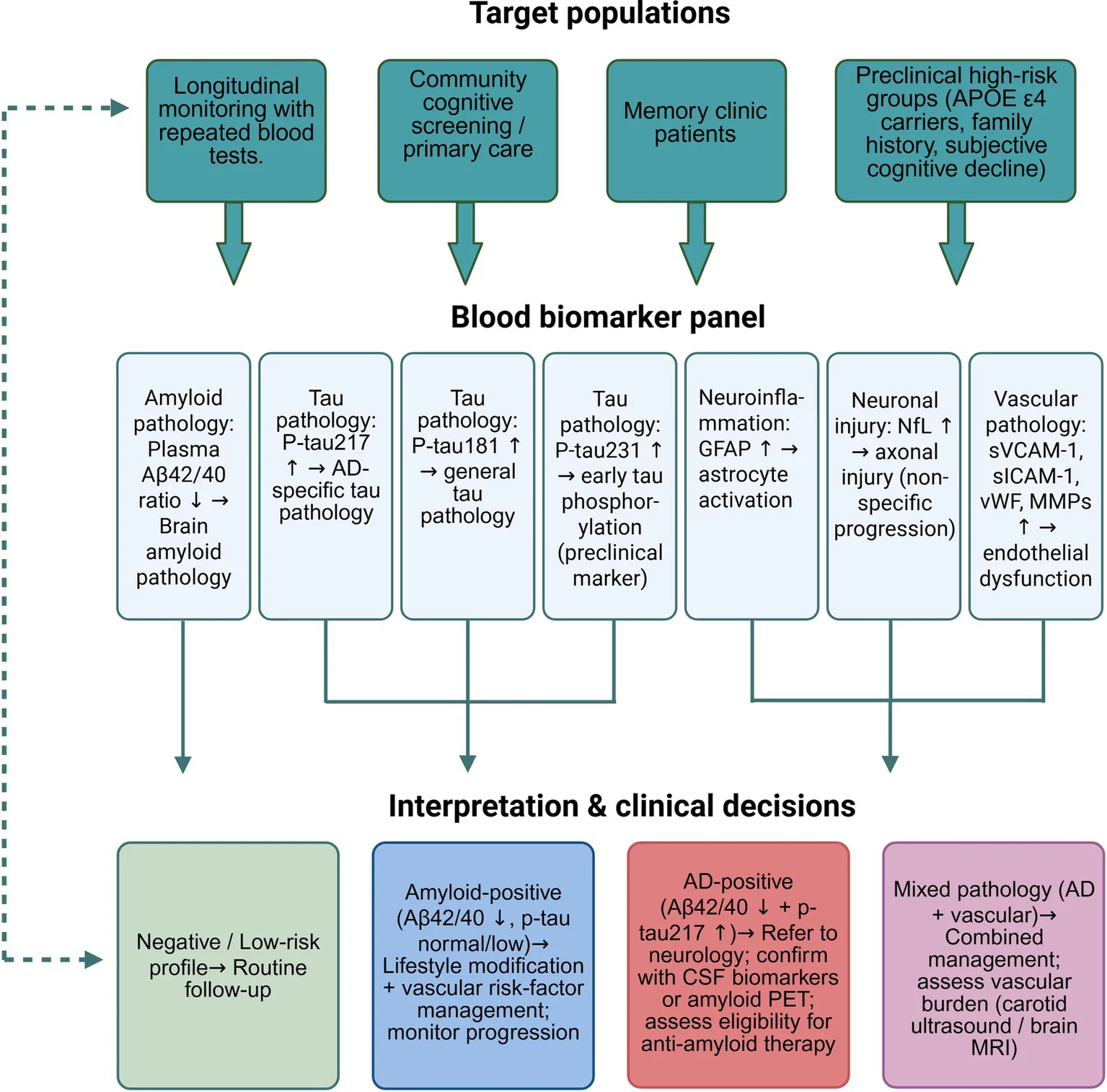
A diagnostic flowchart for blood-based biomarkers in Alzheimer's disease management. This schematic outlines how blood biomarker panels are utilized across various clinical target populations (from primary care to memory clinics and preclinical high-risk cohorts). The biomarker panel assesses amyloidosis, tauopathy, neuroinflammation, neuronal injury, and vascular dysfunction. Based on the specific biomarker combination, the flowchart suggests possible clinical interpretations: a negative or low-risk profile is followed by routine monitoring; an amyloid-positive profile with normal p-tau dictates lifestyle and vascular risk factor management; an AD-positive profile (concomitant amyloid reduction and p-tau elevation) prompts neurological referral and confirmatory testing for anti-amyloid therapy eligibility; and a mixed profile (AD plus vascular pathology) directs comprehensive combined management. APOE genotyping, while valuable for risk stratification, lacks diagnostic sensitivity and specificity for Alzheimer's disease and is therefore used only as a probabilistic modifier alongside core biomarker measurements (Created with BioRender.com). AD, Alzheimer's disease; APOE, apolipoprotein E; Aβ, amyloid-beta; CSF, cerebrospinal fluid; GFAP, glial fibrillary acidic protein; MRI, magnetic resonance imaging; NfL, neurofilament light chain; p-tau, phosphorylated tau; sICAM-1, soluble intercellular adhesion molecule-1; sVCAM-1, soluble vascular cell adhesion molecule-1; vWF, von Willebrand factor; MMPs, matrix metalloproteinases.

## References

[ref1] Altinbas A. van Zandvoort M. J. E. van den Berg E. Jongen L. M. Algra A. Moll F. L. . (2011). Cognition after carotid endarterectomy or stenting: a randomized comparison. Neurology 77, 1084–1090. doi: 10.1212/WNL.0b013e31822e55b9 21880992

[ref2] Anderer S. (2026). FDA approves 2 new Alzheimer disease blood tests. JAMA 336:1197. doi: 10.1001/jama.2026.14721, 42758499.

[ref3] Andjelkovic A. V. Situ M. Citalan-Madrid A. F. Stamatovic S. M. Xiang J. Keep R. F. (2023). Blood-brain barrier dysfunction in normal aging and neurodegeneration: mechanisms, impact, and treatments. Stroke 54, 661–672. doi: 10.1161/STROKEAHA.122.040578, 36848419. PMC9993074

[ref4] Arba F. Vit F. Nesi M. Rinaldi C. Silvestrini M. Inzitari D. (2022). Carotid revascularization and cognitive impairment: the neglected role of cerebral small vessel disease. Neurol. Sci. 43, 139–152. doi: 10.1007/s10072-021-05629-w, 34596778.

[ref5] Arnold S. E. Arvanitakis Z. Macauley-Rambach S. L. Koenig A. M. Wang H.-Y. Ahima R. S. . (2018). Brain insulin resistance in type 2 diabetes and Alzheimer disease: concepts and conundrums. Nat. Rev. Neurol. 14, 168–181. doi: 10.1038/nrneurol.2017.185, 29377010. PMC6098968

[ref6] Ashton N. J. Brum W. S. Di Molfetta G. Benedet A. L. Arslan B. Jonaitis E. . (2024). Diagnostic accuracy of a plasma phosphorylated tau 217 immunoassay for Alzheimer disease pathology. JAMA Neurol. 81, 255–263. doi: 10.1001/jamaneurol.2023.5319, 38252443. PMC10804282

[ref7] Ashton N. J. Janelidze S. Mattsson-Carlgren N. Binette A. P. Strandberg O. Brum W. S. . (2022). Differential roles of Aβ42/40, p-tau231 and p-tau217 for Alzheimer’s trial selection and disease monitoring. Nat. Med. 28, 2555–2562. doi: 10.1038/s41591-022-02074-w, 36456833. PMC9800279

[ref8] Ashton N. J. Pascoal T. A. Karikari T. K. Benedet A. L. Lantero-Rodriguez J. Brinkmalm G. . (2021). Plasma p-tau231: a new biomarker for incipient Alzheimer’s disease pathology. Acta Neuropathol. 141, 709–724. doi: 10.1007/s00401-021-02275-6, 33585983. PMC8043944

[ref9] Baradaran H. Dahlstrom K. A. Culleton S. Sarrami A. H. McFarland M. M. Romero J. R. (2022). Association between extracranial carotid artery plaque and cognitive dysfunction: a systematic review and meta-analysis. Dement. Geriatr. Cogn. Disord. 51, 377–385. doi: 10.1159/000526822, 36316004.

[ref10] Barthélemy N. R. Salvadó G. Schindler S. E. He Y. Janelidze S. Collij L. E. . (2024). Highly accurate blood test for Alzheimer’s disease is similar or superior to clinical cerebrospinal fluid tests. Nat. Med. 30, 1085–1095. doi: 10.1038/s41591-024-02869-z, 38382645. PMC11031399

[ref11] Benedet A. L. Milà-Alomà M. Vrillon A. Ashton N. J. Pascoal T. A. Lussier F. . (2021). Differences between plasma and cerebrospinal fluid glial fibrillary acidic protein levels across the Alzheimer disease continuum. JAMA Neurol. 78, 1471–1483. doi: 10.1001/jamaneurol.2021.3671, 34661615. PMC8524356

[ref12] Biessels G. J. Debette S. Elahi F. M. Geranmayeh F. Hansra G. K. Hughes T. M. . (2026). The vascular contribution to dementia: World Stroke Organization scientific statement. Int. J. Stroke 21, 1211–1234. doi: 10.1177/17474930261470506, 42454751. PMC13672906

[ref13] Brickman A. M. Manly J. J. Honig L. S. Sanchez D. Reyes-Dumeyer D. Lantigua R. A. . (2021). Plasma p-tau181, p-tau217, and other blood-based Alzheimer’s disease biomarkers in a multi-ethnic, community study. Alzheimers Dement. 17, 1353–1364. doi: 10.1002/alz.12301 PMC8451860 33580742

[ref14] Caro I. Western D. Namba S. Sun N. Kawaguchi S. He Y. . (2025). Proteogenomics in cerebrospinal fluid and plasma reveals new biological fingerprint of cerebral small vessel disease. Nat. Aging 5, 2514–2531. doi: 10.1038/s43587-025-01006-w PMC12705447 41266628

[ref15] Chang X.-L. Zhou H.-Q. Lei C.-Y. Wu B. Chen Y.-C. Hao Z.-L. . (2013). Association between asymptomatic carotid stenosis and cognitive function: a systematic review. Neurosci. Biobehav. Rev. 37, 1493–1499. doi: 10.1016/j.neubiorev.2013.05.011, 23727050.

[ref16] Chatterjee P. Pedrini S. Doecke J. D. Thota R. Villemagne V. L. Doré V. . (2023). Plasma Aβ42/40 ratio, p-tau181, GFAP, and NfL across the Alzheimer’s disease continuum: a cross-sectional and longitudinal study in the AIBL cohort. Alzheimers Dement. 19, 1117–1134. doi: 10.1002/alz.12724, 36574591.

[ref17] Chen J.-H. Chang C.-W. Chen Y.-Y. Yang C.-H. Yang W.-B. Hsieh Y.-C. . (2026). Chronic cerebral hypoperfusion exacerbates amyloid and tau pathology by impairing glymphatic transport via AQP4- and VEGF-mediated pathways: insights from a vascular to mixed-type dementia model. Alzheimers Dement. 22:e71290. doi: 10.1002/alz.71290 PMC13093379 41841574

[ref18] Chen F. Zhao J. Meng F. He F. Ni J. Fu Y. (2024). The vascular contribution of apolipoprotein E to Alzheimer’s disease. Brain 147, 2946–2965. doi: 10.1093/brain/awae156 38748848

[ref19] Choi J. D. Moon Y. Kim H.-J. Yim Y. Lee S. Moon W.-J. (2022). Choroid plexus volume and permeability at brain MRI within the Alzheimer disease clinical spectrum. Radiol. Imaging Cancer 304, 635–645. doi: 10.1148/radiol.212400, 35579521.

[ref20] Chun M. Y. Jang H. Kim S.-J. Park Y. H. Yun J. Lockhart S. N. . (2023). Emerging role of vascular burden in AT(N) classification in individuals with Alzheimer’s and concomitant cerebrovascular burdens. J. Neurol. Neurosurg. Psychiatry 95, 44–51. doi: 10.1136/jnnp-2023-331603, 37558399. PMC10803958

[ref21] Cortes-Canteli M. Iadecola C. (2020). Alzheimer’s disease and vascular aging: JACC focus seminar. J. Am. Coll. Cardiol. 75, 942–951. doi: 10.1016/j.jacc.2019.10.062, 32130930. PMC8046164

[ref22] Cozza M. Amadori L. Boccardi V. (2023). Exploring cerebral amyloid angiopathy: insights into pathogenesis, diagnosis, and treatment. J. Neurol. Sci. 454:120866. doi: 10.1016/j.jns.2023.120866, 37931443.

[ref23] de Havenon A. Sheth K. N. Yeatts S. D. Turan T. N. Prabhakaran S. (2022). White matter hyperintensity progression is associated with incident probable dementia or mild cognitive impairment. Stroke Vasc. Neurol. 7, 364–366. doi: 10.1136/svn-2021-001357, 35487617. PMC9453836

[ref24] de la Torre J. C. (2004). Is Alzheimer’s disease a neurodegenerative or a vascular disorder? Data, dogma, and dialectics. Lancet Neurol. 3, 184–190. doi: 10.1016/S1474-4422(04)00683-0 14980533

[ref25] Debette S. Schilling S. Duperron M.-G. Larsson S. C. Markus H. S. (2019). Clinical significance of magnetic resonance imaging markers of vascular brain injury: a systematic review and meta-analysis. JAMA Neurol. 76, 81–94. doi: 10.1001/jamaneurol.2018.3122, 30422209. PMC6439887

[ref26] Duncombe J. Kitamura A. Hase Y. Ihara M. Kalaria R. N. Horsburgh K. (2017). Chronic cerebral hypoperfusion: a key mechanism leading to vascular cognitive impairment and dementia. Closing the translational gap between rodent models and human vascular cognitive impairment and dementia. Clin. Sci. (Lond.) 131, 2451–2468. doi: 10.1042/CS20160727, 28963120.

[ref27] Edwards N. C. Lao P. Alshikho M. J. Mazen J. Huber B. Hale C. . (2025). Alzheimer disease, vascular disease, and blood-brain barrier permeability biomarkers in middle-aged adults. Neurology 105:e214220. doi: 10.1212/WNL.0000000000214220, 41052375. PMC12591055

[ref28] Eisenmenger L. B. Peret A. Famakin B. M. Spahic A. Roberts G. S. Bockholt J. H. . (2023). Vascular contributions to Alzheimer’s disease. Transl. Res. 254, 41–53. doi: 10.1016/j.trsl.2022.12.003, 36529160. PMC10481451

[ref29] Ghaznawi R. Vonk J. M. Zwartbol M. H. Bresser J. de Rissanen I. Hendrikse J. . (2023). Low-grade carotid artery stenosis is associated with progression of brain atrophy and cognitive decline. The SMART-MR study. J. Cereb. Blood Flow. Metab. 43, 309–318. doi: 10.1177/0271678X221133859, 36250500. PMC9903227

[ref30] Gomez G. T. Shi L. Fohner A. E. Chen J. Yang Y. Fornage M. . (2024). Plasma proteome-wide analysis of cerebral small vessel disease identifies novel biomarkers and disease pathways. *medRxiv* [Preprint]. doi: 10.1101/2024.10.07.24314972

[ref31] Gonzalez-Ortiz F. Kac P. R. Brum W. S. Zetterberg H. Blennow K. Karikari T. K. (2023). Plasma phospho-tau in Alzheimer’s disease: towards diagnostic and therapeutic trial applications. Mol. Neurodegener. 18:18. doi: 10.1186/s13024-023-00605-8, 36927491. PMC10022272

[ref32] Graff-Radford J. Simino J. Kantarci K. Mosley T. H. Griswold M. E. Windham B. G. . (2017). Neuroimaging correlates of cerebral microbleeds: the ARIC study (atherosclerosis risk in communities) Stroke 48, 2964–2972. doi: 10.1161/STROKEAHA.117.018336, 29018129. PMC5685663

[ref33] Grande G. Valletta M. R. Rizzuto D. Xia X. Qiu C. Orsini N. . (2025). Blood-based biomarkers of Alzheimer’s disease and incident dementia in the community. Nat. Med. 31, 2027–2035. doi: 10.1038/s41591-025-03605-x, 40140622. PMC12176656

[ref34] Hainsworth A. H. Markus H. S. Schneider J. A. (2024). Cerebral small vessel disease, hypertension, and vascular contributions to cognitive impairment and dementia. Hypertension 81, 75–86. doi: 10.1161/HYPERTENSIONAHA.123.19943, 38044814. PMC10734789

[ref35] Hara S. Seida M. Kumagai K. Yamamoto T. (2020). Beneficial effect of carotid artery stenting on cerebral hemodynamic impairment and cognitive function. Neurol. Med. Chir. (Tokyo) 60, 66–74. doi: 10.2176/nmc.oa.2019-0147, 31748442. PMC7040432

[ref36] He J.-T. Zhao X. Xu L. Mao C.-Y. (2020). Vascular risk factors and Alzheimer’s disease: blood-brain barrier disruption, metabolic syndromes, and molecular links. J. Alzheimers Dis. 73, 39–58. doi: 10.3233/JAD-190764, 31815697.

[ref37] Hong H. Hong L. Luo X. Zeng Q. Li K. Wang S. . (2024). The relationship between amyloid pathology, cerebral small vessel disease, glymphatic dysfunction, and cognition: a study based on Alzheimer’s disease continuum participants. Alzheimer's Res. Ther. 16:43. doi: 10.1186/s13195-024-01407-w, 38378607. PMC10877805

[ref38] Hosoki S. Hansra G. K. Jayasena T. Poljak A. Mather K. A. Catts V. S. . (2023). Molecular biomarkers for vascular cognitive impairment and dementia. Nature Rev. Neurol. 19, 737–753. doi: 10.1038/s41582-023-00884-1 37957261

[ref39] Hurla M. Banaszek N. Kozubski W. Dorszewska J. (2025). Alzheimer’s disease and vascular dementia, connecting and differentiating features. Curr. Alzheimer Res. 22, 2–18. doi: 10.2174/0115672050319219240711103459 39076088

[ref40] Iadecola C. (2013). The pathobiology of vascular dementia. Neuron 80, 844–866. doi: 10.1016/j.neuron.2013.10.008, 24267647. PMC3842016

[ref41] Iadecola C. Gottesman R. F. (2018). Cerebrovascular alterations in Alzheimer disease. Circ. Res. 123, 406–408. doi: 10.1161/CIRCRESAHA.118.313400, 30355253. PMC6214471

[ref42] Iadecola C. Yaffe K. Biller J. Bratzke L. C. Faraci F. M. Gorelick P. B. . (2016). Impact of hypertension on cognitive function: a scientific statement from the American Heart Association. Hypertension 68, e67–e94. doi: 10.1161/HYP.0000000000000053, 27977393. PMC5361411

[ref43] Iliff J. J. Wang M. Liao Y. Plogg B. A. Peng W. Gundersen G. A. . (2012). A paravascular pathway facilitates CSF flow through the brain parenchyma and the clearance of interstitial solutes, including amyloid β. Sci. Transl. Med. 4:147ra111. doi: 10.1126/scitranslmed.3003748, 22896675. PMC3551275

[ref44] Inoue Y. Shue F. Bu G. Kanekiyo T. (2023). Pathophysiology and probable etiology of cerebral small vessel disease in vascular dementia and Alzheimer’s disease. Mol. Neurodegener. 18:46. doi: 10.1186/s13024-023-00640-5, 37434208. PMC10334598

[ref45] Jack C. R. Andrews J. S. Beach T. G. Buracchio T. Dunn B. Graf A. . (2024). Revised criteria for diagnosis and staging of Alzheimer’s disease: Alzheimer’s Association workgroup. Alzheimers Dement. 20, 5143–5169. doi: 10.1002/alz.13859, 38934362. PMC11350039

[ref46] Jack C. R. Bennett D. A. Blennow K. Carrillo M. C. Dunn B. Haeberlein S. B. . (2018). NIA-AA research framework: toward a biological definition of Alzheimer’s disease. Alzheimers Dement. 14, 535–562. doi: 10.1016/j.jalz.2018.02.018 PMC5958625 29653606

[ref47] Jackson R. J. Hyman B. T. Serrano-Pozo A. (2024). Multifaceted roles of APOE in Alzheimer disease. Nature Rev. Neurol. 20, 457–474. doi: 10.1038/s41582-024-00988-2, 38906999. PMC12185264

[ref48] Janelidze S. Bali D. Ashton N. J. Barthélemy N. R. Vanbrabant J. Stoops E. . (2023). Head-to-head comparison of 10 plasma phospho-tau assays in prodromal Alzheimer’s disease. Brain 146, 1592–1601. doi: 10.1093/brain/awac333, 36087307. PMC10115176

[ref49] Janelidze S. Barthélemy N. R. Salvadó G. Schindler S. E. Palmqvist S. Mattsson-Carlgren N. . (2024). Plasma phosphorylated tau 217 and Aβ42/40 to predict early brain Aβ accumulation in people without cognitive impairment. JAMA Neurol. 81, 947–957. doi: 10.1001/jamaneurol.2024.2619, 39068669. PMC11284634

[ref50] Janelidze S. Mattsson N. Stomrud E. Lindberg O. Palmqvist S. Zetterberg H. . (2018). CSF biomarkers of neuroinflammation and cerebrovascular dysfunction in early Alzheimer disease. Neurology 91, e867–e877. doi: 10.1212/WNL.0000000000006082, 30054439. PMC6133624

[ref51] Jung Y. H. Jang H. Park S. B. Choe Y. S. Park Y. Kang S. H. . (2020). Strictly lobar microbleeds reflect amyloid angiopathy regardless of cerebral and cerebellar compartments. Stroke 51, 3600–3607. doi: 10.1161/STROKEAHA.119.028487, 33198580.

[ref52] Kalaria R. N. Sepulveda-Falla D. (2021). Cerebral small vessel disease in sporadic and familial Alzheimer disease. Am. J. Pathol. 191, 1888–1905. doi: 10.1016/j.ajpath.2021.07.004, 34331941. PMC8573679

[ref53] Katusic Z. S. d’ Uscio L. V. He T. (2024). Cerebrovascular endothelial dysfunction: role of BACE1. Arterioscler. Thromb. Vasc. Biol. 44, 1737–1747. doi: 10.1161/ATVBAHA.124.320798, 38868939. PMC11269044

[ref54] Kim H. W. Hong J. Jeon J. C. (2020). Cerebral small vessel disease and Alzheimer’s disease: a review. Front. Neurol. 11:927. doi: 10.3389/fneur.2020.00927, 32982937. PMC7477392

[ref55] Kisler K. Nelson A. R. Montagne A. Zlokovic B. V. (2017). Cerebral blood flow regulation and neurovascular dysfunction in Alzheimer disease. Nature Rev. Neurosci. 18, 419–434. doi: 10.1038/nrn.2017.48, 28515434. PMC5759779

[ref56] Kress B. T. Iliff J. J. Xia M. Wang M. Wei H. S. Zeppenfeld D. . (2014). Impairment of paravascular clearance pathways in the aging brain. Ann. Neurol. 76, 845–861. doi: 10.1002/ana.24271, 25204284. PMC4245362

[ref57] Lazar R. M. Meschia J. F. Edwards L. J. Lal B. K. Howard G. Storm E. . (2026). Revascularization of asymptomatic carotid artery stenosis: cognitive results of the CREST-2 randomized trials. JAMA e2614703. doi: 10.1001/jama.2026.14703, 42747844. PMC13583893

[ref58] Li T. Li D. Wei Q. Shi M. Xiang J. Gao R. . (2023). Dissecting the neurovascular unit in physiology and Alzheimer’s disease: functions, imaging tools and genetic mouse models. Neurobiol. Dis. 181:106114. doi: 10.1016/j.nbd.2023.106114, 37023830.

[ref59] Li Y. Schindler S. E. Bollinger J. G. Ovod V. Mawuenyega K. G. Weiner M. W. . (2022). Validation of plasma amyloid-β 42/40 for detecting Alzheimer disease amyloid plaques. Neurology 98, e688–e699. doi: 10.1212/WNL.0000000000013211, 34906975. PMC8865895

[ref60] Lin Z. Sur S. Liu P. Li Y. Jiang D. Hou X. . (2021). Blood-brain barrier breakdown in relationship to Alzheimer and vascular disease. Ann. Neurol. 90, 227–238. doi: 10.1002/ana.26134, 34041783. PMC8805295

[ref61] Liu Y. Braidy N. Poljak A. Chan D. K. Y. Sachdev P. (2018). Cerebral small vessel disease and the risk of Alzheimer’s disease: a systematic review. Ageing Res. Rev. 47, 41–48. doi: 10.1016/j.arr.2018.06.002 29898422

[ref62] Love S. Miners J. S. (2016). Cerebral hypoperfusion and the energy deficit in Alzheimer’s disease. Brain Pathol. 26, 607–617. doi: 10.1111/bpa.12401, 27327656. PMC8028913

[ref63] Ma Y. Blacker D. Viswanathan A. van Veluw S. J. Bos D. Vernooij M. W. . (2021). Visit-to-visit blood pressure variability, neuropathology, and cognitive decline. Neurology 96, e2812–e2823. doi: 10.1212/WNL.0000000000012065, 33903194. PMC8205457

[ref64] Machaczka O. Skoloudik D. Janoutova J. Roubec M. Reiterova E. Kovalova M. . (2022). Neuropsychological tests and prediction of dementia in association with the degree of carotid stenosis. J. Appl. Biomed. 20, 115–123. doi: 10.32725/jab.2022.018, 36708716.

[ref65] Marshall R. S. Lazar R. M. Meschia J. F. Huston J. Edwards L. J. Brott T. G. . (2026). Revascularization in asymptomatic carotid artery stenosis with hemodynamic impairment and cognitive outcomes: the CREST-H substudy of the CREST-2 randomized clinical trial. JAMA e2613384. doi: 10.1001/jama.2026.13384, 42747834. PMC13583891

[ref66] Mattsson-Carlgren N. Salvadó G. Ashton N. J. Tideman P. Stomrud E. Zetterberg H. . (2023). Prediction of longitudinal cognitive decline in preclinical Alzheimer disease using plasma biomarkers. JAMA Neurol. 80:360. doi: 10.1001/jamaneurol.2022.5272, 36745413. PMC10087054

[ref67] Meyer M. R. Kirmess K. M. Eastwood S. Wente-Roth T. L. Irvin F. Holubasch M. S. . (2024). Clinical validation of the PrecivityAD2 blood test: a mass spectrometry-based test with algorithm combining %p-tau217 and Aβ42/40 ratio to identify presence of brain amyloid. Alzheimers Dement. 20, 3179–3192. doi: 10.1002/alz.13764, 38491912. PMC11095426

[ref68] Milà-Alomà M. Ashton N. J. Shekari M. Salvadó G. Ortiz-Romero P. Montoliu-Gaya L. . (2022). Plasma p-tau231 and p-tau217 as state markers of amyloid-β pathology in preclinical Alzheimer’s disease. Nat. Med. 28, 1797–1801. doi: 10.1038/s41591-022-01925-w, 35953717. PMC9499867

[ref69] Mora P. Laisné M. Bourguignon C. Rouault P. Jaspard-Vinassa B. Maître M. . (2024). Astrocytic DLL4-NOTCH1 signaling pathway promotes neuroinflammation via the IL-6-STAT3 axis. J. Neuroinflammation 21:258. doi: 10.1186/s12974-024-03246-w, 39390606. PMC11468415

[ref70] Ou Y.-N. Tan C.-C. Shen X.-N. Xu W. Hou X.-H. Dong Q. . (2020). Blood pressure and risks of cognitive impairment and dementia: a systematic review and meta-analysis of 209 prospective studies. Hypertension 76, 217–225. doi: 10.1161/HYPERTENSIONAHA.120.14993 32450739

[ref71] Pacholko A. Iadecola C. Iadecola C. Iadecola C. (2024). Hypertension, neurodegeneration, and cognitive decline. Hypertension 81, 991–1007. doi: 10.1161/HYPERTENSIONAHA.123.21356, 38426329. PMC11023809

[ref72] Palmqvist S. Tideman P. Cullen N. Zetterberg H. Blennow K. Alzheimer’s Disease Neuroimaging Initiative (2021). Prediction of future Alzheimer’s disease dementia using plasma phospho-tau combined with other accessible measures. Nat. Med. 27, 1034–1042. doi: 10.1038/s41591-021-01348-z 34031605

[ref73] Pappas C. Bauer C. E. Zachariou V. Libecap T. J. Rodolpho B. Sudduth T. L. . (2025). Synergistic effects of plasma S100B and MRI measures of cerebrovascular disease on cognition in older adults. GeroScience 47, 3131–3146. doi: 10.1007/s11357-024-01498-1, 39907937. PMC12181517

[ref74] Pappas I. Lohman T. Dutt S. Kapoor A. Engstrom A. C. Alitin J. P. M. . (2025). Cerebral hypoperfusion, brain structural integrity, and cognitive impairment in older APOE4 carriers. GeroScience 47, 4973–4986. doi: 10.1007/s11357-025-01642-5, 40220152. PMC12181599

[ref75] Paraskevas K. I. Faggioli G. Ancetti S. Naylor A. R. (2021). Editor’s choice - asymptomatic carotid stenosis and cognitive impairment: a systematic review. Eur. J. Vasc. Endovasc. Surg. 61, 888–899. doi: 10.1016/j.ejvs.2021.03.024, 33966986.

[ref76] Paraskevas K. I. Mikhailidis D. P. Spinelli F. Faggioli G. Saba L. Silvestrini M. . (2023). Asymptomatic carotid stenosis and cognitive impairment. J. Cardiovasc. Surg. (Torino) 64, 167–173. doi: 10.23736/S0021-9509.23.12620-6, 36790142.

[ref77] Perosa V. Oltmer J. Bax F. van den Berg M. L. Auger C. A. Rotta J. . (2025). Relationship between cerebral small vessel disease and proteinopathies in the medial temporal lobe. Acta Neuropathol. Commun. 13:156. doi: 10.1186/s40478-025-02076-y, 40671047. PMC12265290

[ref78] Piegza M. Jaworska I. Piegza J. Bujak K. Dębski P. Leksowska A. . (2022). Cognitive functions after carotid artery stenting-1-year follow-up study. J. Clin. Med. 11:3019. doi: 10.3390/jcm11113019 PMC9180931 35683407

[ref79] Piegza M. Więckiewicz G. Wierzba D. Piegza J. (2021). Cognitive functions in patients after carotid artery revascularization-a narrative review. Brain Sci. 11:1307. doi: 10.3390/brainsci11101307 PMC8533732 34679372

[ref80] Prins N. D. Scheltens P. (2015). White matter hyperintensities, cognitive impairment and dementia: an update. Nat. Rev. Neurol. 11, 157–165. doi: 10.1038/nrneurol.2015.10, 25686760.

[ref81] Raghavan S. Graff-Radford J. Hofrenning E. Fought A. J. Reid R. I. Kamykowski M. G. . (2025). Plasma NfL and GFAP for predicting VCI and related brain changes in community and clinical cohorts. Alzheimer's Dement. 21:e70381. doi: 10.1002/alz.70381, 40566826. PMC12198477

[ref82] Rajeev V. Chai Y. L. Poh L. Selvaraji S. Fann D. Y. Jo D.-G. . (2023). Chronic cerebral hypoperfusion: a critical feature in unravelling the etiology of vascular cognitive impairment. Acta Neuropathol. Commun. 11:93. doi: 10.1186/s40478-023-01590-1, 37309012. PMC10259064

[ref83] Rajeev V. Fann D. Y. Dinh Q. N. Kim H. A. De Silva T. M. Lai M. K. P. . (2022). Pathophysiology of blood brain barrier dysfunction during chronic cerebral hypoperfusion in vascular cognitive impairment. Theranostics 12, 1639–1658. doi: 10.7150/thno.68304, 35198062. PMC8825579

[ref84] Salian V. S. Tang X. Thompson K. J. Curan G. L. Lowe V. J. Li L. . (2024). Molecular mechanisms underlying amyloid beta peptide mediated upregulation of vascular cell adhesion molecule-1 in Alzheimer disease. J. Pharmacol Exp. Ther. 391, 430–440. doi: 10.1124/jpet.124.002280, 39455283. PMC11585316

[ref85] Sanchez E. Coughlan G. T. Wilkinson T. Ramirez J. Mirza S. S. Baril A.-A. . (2025). Association of plasma biomarkers with longitudinal atrophy and microvascular burden on MRI across neurodegenerative and cerebrovascular diseases. Neurology 104:e213438. doi: 10.1212/WNL.0000000000213438, 40063856.

[ref86] Sándor Á. D. Czinege Z. Szabó A. Losoncz E. Tóth K. Mihály Z. . (2024). Cerebrovascular dysregulation and postoperative cognitive alterations after carotid endarterectomy. GeroScience 46, 6301–6315. doi: 10.1007/s11357-024-01237-6, 38877342. PMC11493908

[ref87] Santisteban M. Iadecola C. Carnevale D. Carnevale D. (2023). Hypertension, neurovascular dysfunction, and cognitive impairment. Hypertension 80, 22–34. doi: 10.1161/HYPERTENSIONAHA.122.18085, 36129176. PMC9742151

[ref88] Smith E. E. Aparicio H. J. Gottesman R. F. Goyal M. S. Greenberg S. M. Schneider J. A. . (2025). Vascular contributions to cognitive impairment and dementia in the United States: prevalence and incidence: a scientific statement from the American Heart Association. Stroke 56, e317–e330. doi: 10.1161/STR.0000000000000494, 40820756.

[ref89] Sridharan N. D. Asaadi S. Thirumala P. D. Avgerinos E. D. (2022). A systematic review of cognitive function after carotid endarterectomy in asymptomatic patients. J. Vasc. Surg. 75, 2074–2085. doi: 10.1016/j.jvs.2021.12.059, 34995717.

[ref90] Succar B. Zhou W. (2023). Does carotid intervention improve cognitive function? Adv. Surg. 57, 267–277. doi: 10.1016/j.yasu.2023.04.007 37536858

[ref91] Sun N. Akay L. A. Murdock M. H. Park Y. Galiana-Melendez F. Bubnys A. . (2023). Single-nucleus multiregion transcriptomic analysis of brain vasculature in Alzheimer’s disease. Nat. Neurosci. 26, 970–982. doi: 10.1038/s41593-023-01334-3, 37264161. PMC10464935

[ref92] Sweeney M. D. Ayyadurai S. Zlokovic B. V. (2016). Pericytes of the neurovascular unit: key functions and signaling pathways. Nat. Neurosci. 19, 771–783. doi: 10.1038/nn.4288, 27227366. PMC5745011

[ref93] Sweeney M. D. Sagare A. P. Zlokovic B. V. (2018). Blood-brain barrier breakdown in Alzheimer disease and other neurodegenerative disorders. Nat. Rev. Neurol. 14, 133–150. doi: 10.1038/nrneurol.2017.188, 29377008. PMC5829048

[ref94] Sztriha L. K. Nemeth D. Sefcsik T. Vecsei L. (2009). Carotid stenosis and the cognitive function. J. Neurol. Sci. 283, 36–40. doi: 10.1016/j.jns.2009.02.307 19269651

[ref95] Tarawneh R. (2023). Microvascular contributions to Alzheimer disease pathogenesis: is Alzheimer disease primarily an endotheliopathy? Biomolecules 13:830. doi: 10.3390/biom13050830, 37238700. PMC10216678

[ref96] Tarawneh R. Kasper R. S. Sanford J. Phuah C.-L. Hassenstab J. Cruchaga C. (2022). Vascular endothelial-cadherin as a marker of endothelial injury in preclinical Alzheimer disease. Ann. Clin. Transl. Neurol. 9, 1926–1940. doi: 10.1002/acn3.51685, 36342663. PMC9735377

[ref97] Václavík D. Pakizer D. Hrbáč T. Roubec M. Procházka V. Jonszta T. . (2023). Changes in cognitive functions after carotid endarterectomy and carotid stenting: a decade-apart comparison. Biomedicines 12:13. doi: 10.3390/biomedicines12010013, 38275374. PMC10813376

[ref98] van der Flier W. M. Skoog I. Schneider J. A. Pantoni L. Mok V. Chen C. L. H. . (2018). Vascular cognitive impairment. Nat. Rev. Dis. Primers. 4:18003. doi: 10.1038/nrdp.2018.3, 29446769.

[ref99] van Gennip A. C. E. Satizabal C. L. Tracy R. P. Sigurdsson S. Gudnason V. Launer L. J. . (2024). Associations of plasma NfL, GFAP, and t-tau with cerebral small vessel disease and incident dementia: longitudinal data of the AGES-Reykjavik study. Geroscience 46, 505–516. doi: 10.1007/s11357-023-00888-1, 37530894. PMC10828267

[ref100] Varrias D. Saralidze T. Borkowski P. Pargaonkar S. Spanos M. Bazoukis G. . (2024). Atrial fibrillation and dementia: pathophysiological mechanisms and clinical implications. Biomolecules 14:455. doi: 10.3390/biom14040455, 38672471. PMC11048426

[ref101] Vitali F. Torrandell-Haro G. Arias J. C. French S. R. Zahra S. Brinton R. D. . (2025). Carotid endarterectomy mitigates Alzheimer’s disease and non-Alzheimer’s disease dementia risk linked to asymptomatic carotid stenosis. Alzheimers Dement. 21:e70674. doi: 10.1002/alz.70674 PMC12417316 40922111

[ref102] Viticchi G. Falsetti L. Potente E. Bartolini M. Silvestrini M. (2021). Impact of carotid stenosis on cerebral hemodynamic failure and cognitive impairment progression: a narrative review. Ann. Transl. Med. 9:1209. doi: 10.21037/atm-20-7226, 34430650. PMC8350657

[ref103] Voumvourakis K. Thomaidis N. S. Tsiodras S. (2026). Mapping the brain’s glymphatic system. Biomedicines 14:409. doi: 10.3390/biomedicines14020409, 41751308. PMC12938554

[ref104] Wang J. Huang S. Lan G. Lai Y.-J. Wang Q.-H. Chen Y. . (2025). Diagnostic accuracy of plasma p-tau217/Aβ42 for Alzheimer’s disease in clinical and community cohorts. Alzheimers Dement. 21:e70038. doi: 10.1002/alz.70038 PMC11953589 40156286

[ref105] Wang T. Mei B. Zhang J. (2016). Atherosclerotic carotid stenosis and cognitive function. Clin. Neurol. Neurosurg. 146, 64–70. doi: 10.1016/j.clineuro.2016.03.027 27152468

[ref106] Wardlaw J. M. Smith E. E. Biessels G. J. Cordonnier C. Fazekas F. Frayne R. . (2013). Neuroimaging standards for research into small vessel disease and its contribution to ageing and neurodegeneration. Lancet Neurol. 12, 822–838. doi: 10.1016/S1474-4422(13)70124-8, 23867200. PMC3714437

[ref107] Wierenga C. E. Hays C. C. Zlatar Z. Z. (2014). Cerebral blood flow measured by arterial spin labeling MRI as a preclinical marker of Alzheimer’s disease. J. Alzheimers Dis. 42, S411–S419. doi: 10.3233/JAD-141467, 25159672. PMC5279221

[ref108] Wojdała A. L. Vanbrabant J. Bayoumy S. Antwi-Berko D. Bastard N. L. van der Flier W. M. . (2024). Analytical and clinical performance of eight Simoa® and Lumipulse® assays for automated measurement of plasma p-tau181 and p-tau217. Alzheimer's Res. Ther. 16:266. doi: 10.1186/s13195-024-01630-5, 39702308. PMC11660642

[ref109] Woo M. S. Therriault J. Jonaitis E. M. Wilson R. Langhough R. E. Rahmouni N. . (2024). Identification of late-stage tau accumulation using plasma phospho-tau217. eBioMedicine 109:105413. doi: 10.1016/j.ebiom.2024.105413, 39500009. PMC11570195

[ref110] Wu L.-Y. Chai Y. L. Cheah I. K. Chia R. S. L. Hilal S. Arumugam T. V. . (2024). Blood-based biomarkers of cerebral small vessel disease. Ageing Res. Rev. 95:102247. doi: 10.1016/j.arr.2024.102247, 38417710.

[ref111] Yao M. Liu A. Song J. Xing L. Zi M. Li H. (2026). Cerebral hypoperfusion and the vascular-metabolic-immune-glymphatic network in Alzheimer’s disease: mechanisms, diagnosis, and therapy. Neuroscience 610, 163–188. doi: 10.1016/j.neuroscience.2026.06.001, 42269809.

[ref112] Zenaro E. Piacentino G. Constantin G. (2017). The blood-brain barrier in Alzheimer’s disease. Neurobiol. Dis. 107, 41–56. doi: 10.1016/j.nbd.2016.07.007, 27425887. PMC5600438

[ref113] Zeng X. Lafferty T. K. Sehrawat A. Chen Y. Ferreira P. C. L. Bellaver B. . (2024). Multi-analyte proteomic analysis identifies blood-based neuroinflammation, cerebrovascular and synaptic biomarkers in preclinical Alzheimer’s disease. Mol. Neurodegener. 19:68. doi: 10.1186/s13024-024-00753-5, 39385222. PMC11465638

[ref114] Zlokovic B. V. (2011). Neurovascular pathways to neurodegeneration in Alzheimer’s disease and other disorders. Nat. Rev. Neurosci. 12, 723–738. doi: 10.1038/nrn3114, 22048062. PMC4036520

